# Non-restrictive open vial policy combined with the home visit vaccinations for improving BCG coverage in a high-incidence outreach region: A model-based cost-effectiveness analysis for Indonesia

**DOI:** 10.7189/jogh.13.04049

**Published:** 2023-05-26

**Authors:** Afifah Machlaurin, Jos Luttjeboer, Didik Setiawan, Tjipke Sytse van der Werf, Maarten J. Postma

**Affiliations:** 1 Department of Health Sciences, University Medical Center Groningen, University of Groningen, Netherlands; 2 Department of Clinical and Community Pharmacy, Universitas Jember, Indonesia; 3 Asc Academics, Groningen, Netherlands; 4 Faculty of Pharmacy, Universitas Muhammadiyah Purwokerto, Indonesia; 5 Department of Pulmonary Diseases & Tuberculosis, University Medical Center Groningen, Netherlands; 6 Department of Economics, Econometrics & Finance, Faculty of Economics & Business, University of Groningen, Netherlands; 7 Department of Pharmacology & Therapy, Universitas Airlangga, Indonesia; 8 Center of Excellence in Higher Education for Pharmaceutical Care Innovation, Universitas Padjadjaran, Indonesia

## Abstract

**Background:**

Bacillus Calmette-Guérin (BCG) vaccination is recommended at birth or in the first week of life to achieve the most beneficial effects in protecting against the most severe type of tuberculosis (TB) disease in children. However, delayed vaccination is commonly reported, especially in outreach or rural areas. We assessed the cost-effectiveness of combining non-restrictive open vial and home visit vaccination strategies in order to increase timely BCG vaccination in a high-incidence outreach setting.

**Methods:**

We applied a simplified Markov model for the Papua setting, which resembled a high-incidence outreach setting in Indonesia, to assess the cost-effectiveness of these strategies from a health care and a societal perspective. A moderate increase (75% wastage rate and 25% home vaccination) and a large increase (95% wastage rate and 75% home vaccination) scenario were assessed in the analysis. We calculated incremental cost-effectiveness ratios (ICER) based on the incremental costs and quality-adjusted life years (QALYs) gained by comparing the two strategies to the base case scenario (35% wastage rate and no home vaccination).

**Results:**

The costs per vaccinated child were US$10.25 in the base case scenario, increasing slightly in the moderate (US$10.54) and large increase scenarios (US$12.38). The moderate increase scenario was predicted to prevent 5783 TB-related deaths and 790 TB cases while the large increase scenario predicted the prevention of 9865 TB-related deaths and 1348 TB cases for the entire lifespan of our cohort. From a health care perspective, the ICERs were predicted to be US$288/QALY and US$487/QALY, respectively, for the moderate and large increase scenarios. Using Indonesia’s gross domestic product (GDP) per person as a threshold, both strategies were considered to be cost-effective.

**Conclusions:**

We found that the allocation of resources for timely BCG vaccination based on combining home vaccination and a less restrictive open vial strategy could substantially reduce childhood TB cases and TB-related mortality. Although outreach activities are more expensive than vaccination at a health care facility only, these activities proved to be cost-effective. These strategies might also be beneficial in other high-incidence outreach settings.

The Bacille Calmette–Guérin (BCG) vaccine was introduced decades ago and remains the only available tuberculosis (TB) vaccine on the market. The World Health Organization (WHO) recommends a universal vaccination strategy by vaccinating all infants in high TB incidence countries [1] at birth or in their first week of life, as this prevents infants from contracting the most severe type of TB [2]. Timely vaccination at birth is reported to have the most beneficial effects on reducing TB-related death in children compared to a later schedule, eg, six weeks after birth or at the recommended age of the first dose of diphtheria, tetanus, and pertussis (DTP) vaccination [3]. In fact, delayed BCG vaccination is commonly reported in low-middle income countries (LMICs), where administration may be delayed by up to 10 weeks [4].

In Indonesia, BCG vaccination has been included in the national immunisation programme since 1956 [5]; in 2017, its coverage was high, at an average of 87% [6]. Despite this high vaccination coverage, Indonesia’s TB incidence remains the third-highest globally [7]. This can be partly explained by the substantially lower vaccination coverage among within first two months of life, at almost half compared to the national coverage [4,8,9]. Furthermore, coverage differs between regions, from 69.8% in Papua to 98.9% in Yogyakarta [6]. There are several context-specific reasons why the coverage is very low in some settings, especially in outreach or rural areas such as Papua. Children who live in urban areas or near a health care facility are less likely to miss their BCG vaccination as adherence to BCG vaccination is influenced by the children’s area of residence, rather than the mother’s education and knowledge [10,11]. Mothers who have more frequent health care visits show a higher BCG vaccination uptake for their infants. Children born at home are at an increased risk of being vaccinated later than recommended or of not being vaccinated at all [11]. To minimise the wastage of 20-dose BCG vaccines, the vaccine may be delivered less frequently in the outreach region. The health care worker tends to wait for enough infants to be vaccinated to decrease costs, which might reduce vaccination uptake.

As BCG vaccination is one of the cornerstones of the TB prevention programme, efforts to increase the BCG vaccination coverage are important, especially in high-incidence rural and remote areas. The measles vaccination case showed that encouraging health care workers to open a vial regardless of the number of children to be vaccinated and promoting home visits in rural areas improved vaccination coverage [12]. Following WHO guidelines to open multidose vials despite any wastage of unused vaccine [1], these strategies might be warranted in outreach areas to reduce missed BCG vaccination at birth.

To achieve and maintain high vaccination coverage in rural, remote areas, additional investments in the national immunisation programme are needed, since current funds and efforts are not sufficient to provide all children in Indonesia with a BCG vaccine. Given its potentials to enhance health gains in Indonesia, we considered the implementation of a non-restrictive open vial policy combined with pro-active home visits by health care workers for improving the vaccination coverage. We aimed to evaluate the cost-effectiveness of the combined strategy in different scenarios, by calculating the costs required and the health impacts in high-incidence outreach settings such as Papua.

## METHODS

### Cost-effectiveness analysis

This study is a model-based cost-effectiveness analysis, performed from both a health care and a societal perspective. We calculated the costs required for the implementation of a non-restrictive open vial use strategy, combined with performing home visit vaccinations. We calculated the health impacts of the improved vaccination coverage on the number of tuberculosis cases and deaths, as well as the quality-adjusted life-years (QALYs) gained over a lifespan with an average life expectancy of 70 years. We calculated both direct medical costs and lost-productivity costs incurred by TB patients during the treatment and disease stages. We evaluated the incremental cost-effectiveness ratio (ICER) based on the incremental costs per incremental QALY for context-specific vaccination coverages (moderate increase scenario and large increase scenario) compared to the base of current coverage (**Table 1**). We applied once gross domestic product (GDP) per person in 2020 (US$3871 per year) as the willingness-to-pay threshold. A scenario is considered cost-effective if the ICER is below this threshold. We ran the calculation and model simulation in the Microsoft Excel 2016. An annual timestep and a 3% discount rate for both the costs and the outcomes were applied in the model [23].

**Table 1:** Input parameters and assumptions with respective distributions for probabilistic sensitivity analysis

Parameter	Point of estimates	Distribution (interval*)	Reference
One birth cohort	170 100	Fixed	Kesehatan et al. [6]
Incidence	0.6%	Fixed	Kesehatan et al. [6]
RR TB disease in latent TB population	21.00%	Log normal (14%-30%)	Andrews et al. [13]
Case detection rate	53.00%	Beta (48%-58%)	World Health Organization [14]
Percentage MDR/RRTB	2.74%	Beta (1.90%-3.69%)	World Health Organization [14]
Mortality rate (yearly)			
*Healthy population*	Age-stratified	Fixed	The World Bank [15]
*Latent TB*	0.12%	Beta (0.07%-0.17%)	Miller et al. [16]
Untreated patient outcomes			Tiemersma et al. [17] calculated
*Death*	0.11	Dirichlet	
*Recovery without treatment*	0.03	Dirichlet	
Treatment outcomes (%)			World Health Organization [14] calculated
DSTB treatment			
*Success*	85.77%	Dirichlet	World Health Organization [14]
*Failure*	0.38%	Dirichlet	World Health Organization [14]
*Death*	2.46%	Dirichlet	World Health Organization [14]
*Lost to follow-up*	5.38%	Dirichlet	World Health Organization [14]
MDR/RRTB treatment			
*Success*	47.28%	Dirichlet	World Health Organization [14]
*Failure*	3.71%	Dirichlet	World Health Organization [14]
*Death*	16.17%	Dirichlet	World Health Organization [14]
*Lost to follow-up*	31.12%	Dirichlet	World Health Organization [14]
Vaccine efficacy			
TB infection			
*TB disease*	71%	Beta (42%-85%)	Roy et al. [18]
*Progression from TB infection to disease*	58%	Beta (23%-77%)	Roy et al. [18]
*Duration of protection (years)*	10		
*Waning effects*	no		
Costs of treatment			
*DSTB treatment (US$)*	35	Gamma (SD = 25%)	World Health Organization [19]
*MDR/RRTB treatment (US$)*	1296	Gamma (SD = 25%)	World Health Organization [19]
Productivity losses			
*DSTB (days)*	25	Normal (SD = 25%)	Collins et al. [20]
*MDR/RRTB (days)*	102	Normal (SD = 25%)	Collins et al. [20]
Minimum wage per day (US$)	4.82	Gamma (4.70-4.95)	Badan Prusat Statistik [21]
Utility			
*Healthy*	1.00		Assumed
*Latent TB*	0.82	Beta (0.80-0.85)	Guo et al. [22]
*Active TB*	0.68	Beta (0.65-0.72)	Guo et al. [22]
*Treatment*	0.68	Beta (0.65-0.72)	Assumed
Discount rate (costs and utility)	3%		Walker et al. [23]

TB – tuberculosis, RR – relative risk, DSTB – drug-susceptible tuberculosis, MDR/RRTB – multidrug-resistance/rifampicin-resistance TB, SD – standard deviation

*Interval reflected as the lower and upper values; we used 95% CIs where applicable or SD = 25% to the point of estimate in the base case. We applied specific distributions to the central estimates in the probabilistic sensitivity analysis.

### Model setting

**Figure 1:**
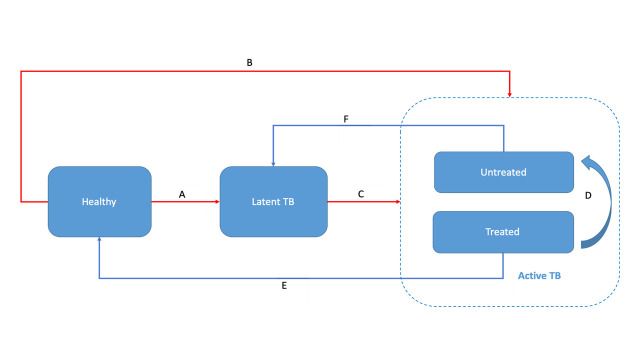
Natural course of tuberculosis (TB). The model was based on age-stratified incidence and implicitly included mortality rate in each group. A = infection, B = fast progression to active TB, C = slow progression to active TB, D = treatment failure, E = successful treatment, F = self-healing. Red line is affected by vaccine effectiveness.

We followed a published simplified Markov model [24] to assess the costs and health impacts of increasing the BCG vaccine coverage in outreach area settings by allowing moderate to non-restrictive open vial strategies and promoting home visit vaccination (**Figure 1**). The model simulates one birth cohort that comprises susceptible healthy new-borns. Following infection, newborns can develop TB directly (active TB compartment) or enter a latency phase (latent TB compartment). In the active TB compartment, individuals are divided into a treated and an untreated group. The treated group is further subdivided into a drug-susceptible TB (DSTB) group and a rifampicin and/or multidrug resistant TB (RRTB and/or MDRTB) group, both of which encounter specified treatment costs and outcomes. The untreated group comprises patients with active TB who have not yet been diagnosed. Untreated patients may recover without treatment, but do not necessarily clear the infection and are assumed to move to the latent TB compartment. Individuals with latent TB are at a reduced risk of developing active TB, with a relative risk (RR) of 21% (95% confidence interval (CI) = 14%-30%)) [13] compared to healthy unvaccinated individuals. There are four treatment outcomes; success, failure, death, and lost to follow-up. Successful treatment outcome brings patients into the healthy group, since these individuals have cleared their infection. Failed treatment results in patients remaining in the group with active TB, as if untreated. Each group was assigned a specific mortality rate (**Table 1**), which was age-stratified for the healthy population and fixed for others.

### Data and model assumption

#### Population setting

We engaged with the Indonesian national TB programme and applied the model to one of its provinces, Papua, which reflects a high-incidence outreach setting in Indonesia. We retrieved the birth cohort size of Papua from the Indonesian Basic Health Surveillance data (RISKESDAS, 2018 data) [25] and used it as the initial population of the model. We simulated the cohort over a lifespan with an average life expectancy of 70 years (Indeks pembangunan manusia 2018, BPS) [21]. In 2018, the reported BCG vaccination coverage for Papua was 69.8% and this was used as a base case coverage [25]. We applied the 2018 TB incidence in Papua (0.6%) and assumed that the incidence was the same for all ages [25].

#### Vaccine characteristics

We derived BCG vaccine effectiveness from a meta-analysis study, which included 14 studies worldwide, to be 27% (RR = 0.73, 95% CI = 0.61-0.87) and 71% (RR = 0.29, 95% CI = 0.15-0.58) against infection and active TB, respectively. Additionally, the vaccine was expected to provide protection against progression from infection to active disease at 58% (RR = 0.42, 95% CI = 0.23-0.77) [18]. Regarding the waning effect of the BCG vaccine, we assumed protection up to 10 years, as the study showed that the most significant vaccine effectiveness of BCG happen from zero to nine years [26]. We applied 87% vaccine coverage for the base case.

#### Costs of vaccination

Since the vaccine delivery costs for rural and urban areas are uncertain, we defined a composite of vaccination costs per infant that includes vaccine costs, wastage, freight and supply, administration, immunization support, and programme-related costs. We assumed that the costs for BCG vaccination per child in rural areas, which we considered remote areas, required greater vaccine vial, syringe, and safety box supplies. Additionally, the health care staff would spend extra time on vaccination and transportation to reach the areas to actively vaccinate new-borns at home.

The cost components of vaccination are described in **Table 2**. We derived the vaccine costs from the 2020 UNICEF price for a 20-dose BCG vial at US$2.10 [27]. We used the 2020 UNICEF negotiated price of US$0.057 per syringe and US$0.54 for one safety box, assuming one safety box per 100 children vaccinated. The WHO estimates freight costs, including insurance, at 6% of the vaccine price and 15% of the supplies costs [28]. However, for remote areas, we assumed a 10% higher cost for vaccines and 20% for supplies. For the extra health care workload, we estimated the cost at US$10 (range US$8-12) per infant vaccinated based on the price list of average BCG vaccination services at private clinics in urban areas in Indonesia. Extra transportation costs were applied when the health care staff needed to vaccinate the infants at home.

**Table 2:** Cost components of BCG vaccination

Cost components	Base case	Distribution (interval)*	Reference
Vaccine costs per vial (20-dose vial)	US$2.10	Gamma (1.90-5.38)	UNICEF Supply Division [27]
Syringe costs per vaccinated child	US$0.057	Gamma (SD = 25%)	UNICEF Supply Division [27]
Safety box costs per 100 vaccinated children	US$0.54	Gamma (SD = 25%)	UNICEF Supply Division [27]
Freight costs for vaccine	10%	Fixed	assumed
Freight costs for supplies	20%	Fixed	assumed
Costs of administration and delivery per vaccinated child	US$10	Gamma (8-12)	assumed
Costs of transportation per vaccinated child	US$5	Gamma (SD = 25%)	assumed

SD – standard deviation

*Interval reflected as the lower and upper values; we used 95% CIs where applicable or SD = 25% to the point of estimate in the base case. We applied specific distributions to the central estimates in the probabilistic sensitivity analysis.

### Scenarios

#### Base case

The base case resembles the current practice in Papua. We applied a 70% vaccination coverage based on the current situation of BCG vaccination in Papua [6]. We assumed a 35% wastage rate for a vaccine vial containing 20 doses, since there are no country-specific data for BCG vaccination wastage in rural areas. This estimate was based on several studies: a study in the national hospital setting in Guinea-Bissau showed that the median wastage rate for BCG was 35% [29]; a study found that the wastage rate in the Gambia was 18.5%-79.0% [30], while another found it to be 35.1% and 37.1% in two districts in India [31]; in Bangladesh, higher wastage rates were reported for the BCG vaccine (55%-93%) [32]. Vaccine wastage was calculated by the following formula: (vaccine doses supplied − vaccine doses used)/vaccine doses supplied. In the base case scenario, we assumed all vaccinations were administered at a health care centre.

#### Two scenarios proposed

Two scenarios are analysed in this study. First, the large increase scenario was considered as a high vaccination coverage up to 99% by allowing even a 95% vaccine wastage rate and 75% home visit vaccinations. This wastage rate corresponds to a situation in which the health care worker could open a 20-dose BCG vial for one eligible infant. Second, the moderate increase scenario assumed a moderate vaccination coverage up to the national level of coverage at 87%, allowing 75% vaccine wastage and implementing 25% home visit vaccinations (**Table 3**). The assumption for the large increase scenario was motivated by a study of measles vaccination, which showed that home visits after delivery and removing the wastage restriction could increase the vaccination coverage to up to 97% [33]. The moderate increase scenario lay between the status quo and the large increase scenario which resembled as the national vaccination coverage. The wastage rates for the two scenarios were based on the WHO tool for estimating the wastage of BCG vaccination for Indonesia, where the predicted BCG wastage in 2020 was 83% [34]. For these strategies, the wastage rate was assumed around the range between 75% and 95% for the moderate and the large increase scenario, respectively. The large increase scenario has a higher wastage rate since the number of home visits is higher than in the moderate increase scenario, meaning more vials will be opened regardless of the number of newborns requiring vaccination.

**Table 3:** Summary of the scenarios explored in the model

Strategy	Base case (status quo)	Moderate increase scenario	Large increase scenario
Vaccine wastage	35%	75%	95%
Home visit vaccination	0	25%	75%
Vaccine coverage	70%	87%	99%

#### Sensitivity analysis

We performed a univariate sensitivity analysis to investigate the key drivers of the cost-effectiveness analysis by varying the base case values with lower and upper values of each parameter. The likely range of the parameters was based on the CIs, and a standard deviation (SD) = 25% was used when there was no CI or other plausible range available.

To account for uncertainty in the deterministic outcomes of the model, we performed a probabilistic sensitivity analysis (PSA). The parameters’ ranges were used to assign a distribution to most parameters (**Table 1**). The costs per vaccinated child were uniformly distributed with a range of SD = 25% of the point estimate. We conducted a Monte Carlo simulation by running the model over 1000 iterations, each iteration using random parameter values drawn from the respective parameter distribution. We used the same ranges to assign distributions to the parameters. The PSA results were presented in a cost-effectiveness acceptability curve (CEAC), using the threshold to predict the probability of the strategy being cost-effective.

## RESULTS

Based on our vaccination costs calculation, in the base case scenario, the costs per vaccinated child were US$10.25, in line with the current vaccination price in Indonesia. Targeting a 75% wastage rate and 25% home visit vaccinations for a moderate increase scenario, the costs per vaccinated child were slightly increased at US$10.54. In the large increase scenario, which used a conservative wastage rate of 95% and assumed to reach 75% of children who did not have access to a health care facility, the costs per vaccinated child were increased to US$12.38. Regarding costs, the large increase scenario requires more costs compared to the moderate increase scenario due to the higher home visit costs and the wastage of BCG vaccine. The incremental vaccination costs were estimated to be US$540 818 for the moderate and US$1 266 576 for the large scenario (**Table 4**). Expectedly, the ICER in the large increase scenario was higher than in the moderate increase scenario. The ICERs from the health care perspective were predicted to be US$288/QALY for the moderate and US$487/QALY for the large increase scenario. From a societal perspective, when productivity losses are incorporated in the estimations, the ICERs for the two scenarios were slightly improved, at US$278/QALY and US$477/QALY, respectively. Although the ICER for the large increase scenario was almost twice that of the moderate increase scenario, if the decision-maker sets a willingness-to-pay of once GDP per capita (US$3871 per year in 2020), both strategies are considered cost-effective and justified to be applied in high-incidence outreach areas.

**Table 4:** BCG vaccination cost components based on the BCG vaccination scenarios

Cost components (US$)*	Base case scenario	Moderate increase scenario	Large increase scenario
BCG vaccine vial costs	18 960	62 155	321 489
Syringe costs	6690	8435	8726
Safety box costs	634	799	827
Freight costs for vaccine	1896	6215	32 149
Freight costs for supplies	879	1108	1146
Administration and delivery costs	1 173 690	1 479 870	1 530 900
Transportation costs	0	184 984	574 088
Total vaccination costs	1 202 748	1 743 566	2 469 325
*Costs per vaccinated child*	10.25	11.78	16.13
*Incremental costs of vaccination per birth cohort*		540 818	1 266 576

*Costs were presented in 2020 exchange rates for US$.

The vaccination strategies were estimated to have more impact on preventing TB cases in childhood compared to later age (**Table 5**). The moderate increase scenario could prevent 790 TB cases, while the large increase scenario could prevent 1348 cases. Compared to the base case scenario, the moderate increase scenario was predicted to prevent 569 childhood TB-related deaths during the first 15 years, while the large increase scenario was predicted to prevent almost twice that number at 971 TB-related deaths. Over the entire lifespan of the cohort, the vaccination strategies were predicted to prevent 5783 and 9865 TB-related deaths in the moderate and large increase scenarios, respectively.

**Table 5:** Estimated costs and outcomes per birth cohort according to the BCG vaccination scenarios

Costs and outcomes per birth cohort*	Base case scenario	Moderate increase scenario	Large increase scenario
**Costs in US$**			
Total costs from the health care perspective	1 339 682	1 852 423	2 817 599
Total costs from the societal perspective	1 540 326	2 035 195	2 987 755
Incremental costs from the health care perspective		512 741	1 477 918
Incremental costs from the societal perspective		494 869	1 447 429
**Outcomes**			
QALYs	4 295 720	4 297 498	4 298 753
LYG	8 198 126	8 200 950	8 202 944
Incremental LYG		2824	4817
Incremental QALYs		1778	3033
Cases prevented in the first five years of life		209	357
Cases prevented in the first 15 y of life		790	1348
Cases prevented in the first 30 y of life		956	1630
Cases prevented in the first 50 y of life		967	1649
Deaths prevented in the first five years of life		42	71
Deaths prevented in the first 15 y of life		536	915
Deaths prevented in the first 30 y of life		1707	2912
Deaths prevented in the first 50 y of life		2894	4936
TB-related deaths prevented in the first five years of life		45	77
TB-related deaths prevented in the first 15 y of life		569	971
TB-related deaths prevented in the first 30 y of life		1928	3289
TB-related deaths prevented in the first 50 y of life		3867	6596
Total TB-related deaths prevented		5783	9865
**ICER per QALYs**			
*Healthcare perspective*		288	487
*Societal perspective*		278	477

QALY – quality adjusted life year, LYG – life year gained, ICER – incremental cost-effectiveness ratio, TB – tuberculosis

*Costs were presented in 2020 in US$.

**Figure 2:**
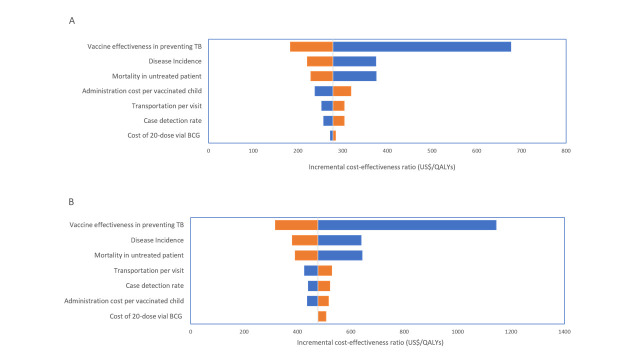
Univariate sensitivity analysis from a societal perspective. **Panel A.** Moderate increase scenario. **Panel B.** Large increase scenario.

The one-way sensitivity analysis from a societal perspective is presented in the tornado diagram in **Figure 2**. The top three key drivers of the analysis of both scenarios were vaccine effectiveness against TB disease, incidence, and mortality in untreated patients. The fourth most influential parameter varied between moderate and large increase scenario; administration costs for the moderate increase scenario and transportation costs for the large increase scenario.

**Figure 3:**
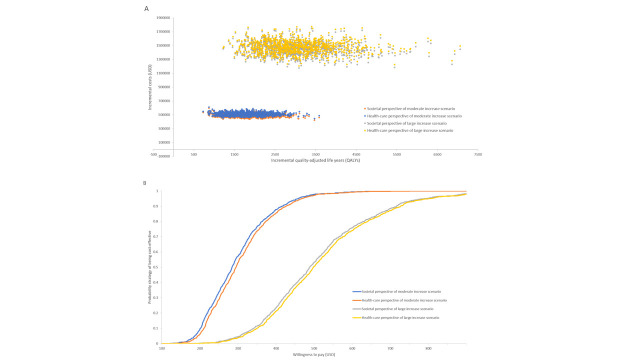
Probabilistic sensitivity analysis: a) cost-effectiveness plane and b) cost-effectiveness acceptability curve (1000 iterations).

The PSA showed that using one GDP per person (US$3871 per year in 2020) as the willingness-to-pay threshold, both moderate and large increase scenarios are cost-effective. The corresponding scatter plot and cost-effectiveness acceptability curves are presented in **Figure 3**. The cost-effectiveness acceptability curves suggest that applying lower willingness-to-pay, corresponding to a monthly salary in Indonesia of US$500/QALY, the probability of the moderate increase scenario being cost-effective was predicted to be 97%, while the probability for the large increase scenario was predicted to be only around 50%.

## DISCUSSION

### Main findings

In rural, remote areas such as the Papua setting, we estimated that increasing the coverage of BCG vaccination was beneficial in reducing TB cases (especially in childhood) and overall TB-related mortality. In our moderate increase scenario, increasing local coverage in Papua (69%) up to the national level (87%) by allowing higher vaccine wastage to 75% (compared to the base case scenario of 35%) and reaching 25% of children who have no access to a health care facility through home vaccination (rather than only vaccinating children at the health care centre as is the current situation) would prevent 790 TB cases during the first 15 years in one birth cohort. More substantial health benefits could be achieved by implementing the large increase scenario, which allows a more active vaccination strategy by combining 75% home vaccination and a non-restrictive open vial strategy. Compared to the moderate increase scenario, the large increase scenario was predicted to prevent almost twice the number of TB cases in childhood and TB-related mortality. The WHO has recently paid more attention to childhood TB burden to better understand the impact of BCG vaccination. Additionally, TB-related deaths remain a significant concern, with 1.2 million TB-related deaths in human immunodeficiency virus (HIV) negative individuals in 2018. Reflecting on the WHO 2020 milestone of a 20% reduction in TB incidence and a 35% reduction in TB-related deaths, Indonesia was still off target. Increased coverage may indeed emerge especially in rural or hard to reach areas. This could be achieved by lifting the open vial restriction and reaching children who have no access to a health care centre through home visit vaccinations. We showed that in rural settings such as Papua, these two cost-effective policy adjustments may substantially reduce the number of childhood TB cases and TB-related deaths.

Besides a lack of visits to a health care facility, parental attitudes and a lack of knowledge might hinder BCG vaccine coverage of newborns in rural areas [8]. Home visits may help with overcoming these barriers by providing BCG vaccines on time and increasing awareness among parents by informing them about the benefits of vaccination. Home visit vaccinations might require some investments from the government, especially for transportation and administration. However, this visit can be combined with other health care programmes, such as other newborn vaccination programmes or the postnatal check-up.

Although home vaccination and a less restrictive open vial strategy produce a higher ICER compared to the current vaccination strategy in rural area settings with a high incidence of TB, these strategies are considered very cost-effective.

### Strengths and limitations

This study has several strengths. To our knowledge, it is the first to explore the cost-effectiveness of combining the implementation of home visit vaccinations and less restrictive multidose open vial strategies in the BCG vaccination programme in rural areas. These strategies were determined to increase the coverage of timely BCG vaccination, preferably at birth, to reduce TB cases and TB-related deaths. Second, we applied extensive sensitivity analyses to assess the impact of uncertainty in the input parameters on the outcome and further explored the key variables that influence the results. Our PSA showed that, even at a lower threshold of US$500/QALY, the probability of both strategies being cost-effective was 97%. Beyond clinical efficacy inputs, the one-way sensitivity analysis identified the costs of administration and transportation as important drivers. Therefore, the government could optimize these cost variables to achieve optimum beneficial impacts regarding investments. Third, our model could be used for any data setup, so our results could be updated and validated when more robust evidence emerges; for example, concerning the effectiveness of home visit vaccination and the implementation of a non-restrictive open vial strategy to increase vaccine coverage, or the effectiveness of the BCG vaccine in high incidence areas.

As in any model-based analysis, our study is subject to limitations, especially regarding the uncertainties of the input parameters and assumptions. First, there is the absence of age-stratified incidence data in Papua. We applied a uniform incidence retrieved from the local surveillance data for all ages. As disease incidence was the second key driver of the analysis, under- or over-estimation of this variable might influence the results and, in a worst-case scenario, even change the recommendation. However, in a high incidence country such as Indonesia, the incidence is likely to be underreported [35], which makes our analysis conservative.

## CONCLUSIONS

Using a modelling approach, we showed that allocation of resources for timely home vaccination of children without or with poor access to a health care facility combined with a less restrictive open vial strategy could substantially reduce childhood TB cases and TB-related mortality. Although outreach activities are more costly than vaccination at a health care facility only, these activities proved to be cost-effective. The cost-effectiveness profile might become even more favourable when home BCG vaccination is combined with other vaccinations or a postnatal check-up.
